# Relationships of deworming drug consumption and animal protein intake with stunting

**DOI:** 10.1016/j.parepi.2023.e00326

**Published:** 2023-10-05

**Authors:** Dessy Hermawan, Devi Kurniasari, Vira Sandayanti, Nurhalina Sari, Erna Listyaningsih

**Affiliations:** a Department of Nursing, Faculty of Health Sciences, Malahayati University, Bandar Lampung, Indonesia; b Department of Midwifery, Faculty of Health Sciences, Malahayati University, Bandar Lampung, Indonesia; c Department of Psychology, Faculty of Health Sciences, Malahayati University, Bandar Lampung, Indonesia; d Department of Public Health, Faculty of Health Sciences, Malahayati University, Bandar Lampung, Indonesia; e Department of Management, Faculty of Economics, Malahayati University, Bandar Lampung, Indonesia

**Keywords:** Stunting, Deworming drug, Animal protein

## Abstract

By the end of 2022, the nationwide incidence of stunting remained high, including in Lampung Province, where it was 15.8%, above the target of 14% to be achieved by 2024. Since 2019, stunting has become a national priority due to suspected factors such as low nutrition intake, especially from animal protein sources, high rates of worm infections, and low compliance of under-fives in consuming deworming drugs. Therefore, this research aimed to analyze the relationship between deworming consumption, adequacy of animal protein intake, and stunting incidence in children aged 12–59 months in Bandar Lampung in 2022.

This research used an analytic survey with a cross-sectional approach to analyze the relationship between deworming consumption, adequacy of animal protein intake, and stunting in under-fives at two priority stunting handling neighbourhoods in Bandar Lampung, namely Way Gubak and Karang Maritim. The sample consisted of 262 under fives and their mothers who visited the integrated health post in the selected neighbourhoods from November to December 2022, and the data obtained were analyzed using logistic regression.

The results revealed a relationship between the consumption of an deworming and varying animal protein consumption with stunting incidence. This indicated a need to promote the importance of consuming deworming and adequate consumption of animal protein with variation to prevent stunting in children in Bandar Lampung.

## 1 Introduction

Stunting is a national problem in Indonesia, where children experience growth failure. According to the Indonesian Nutritional Status Study/SSGI conducted by the Ministry of Health in 2022, the national stunting rate is 21.6% (Ministry of Health of the Republic of Indonesia, 2023). Although the condition has decreased compared to 2021 at 24.4% (Ministry of Health of the Republic of Indonesia, 2021), the decline has yet to reach the national target of below 14% (Ministry of State Apparatus Empowerment and Bureaucratic Reform of the Republic of Indonesia, 2022). Therefore, serious efforts from all existing components are still needed to overcome the stunting problem in the country.

Stunting can negatively impact children in the future, including short-term and long-term effects (Soliman et al., 2021). Besides causing children to be short and prone to illness, this condition can also affect children's cognitive or intellectual development (Ekholuenetale et al., 2020). Children with stunting tend to have low intellectual abilities, which will affect the nation's competitiveness in the future.

Until early 2023, several parties have tried to achieve the target of reducing the stunting rate below 14% in 2024. These interventions ranged from improving the stunting case recording system (Khasanah et al., 2022) to focusing on pregnant women and children in the first 1000 days of life by providing specific and sensitive nutrition interventions (Candriasih et al., 2021; Hafid et al., 2021). However, the reduction in stunting rates has yet to fulfil expectations. Currently, the focus widely discussed and implemented is the provision of animal protein intake because intake of animal-based foods is strongly associated with stunting incidence (Headey et al., 2018). This condition is exacerbated by the low economic ability of the community due to the post-pandemic effect, which reduced the community's purchasing power.

Adequate food access and intake do not guarantee that children will be free from stunting, as other factors, such as parasitic infections through worms in the intestine, can interfere with nutrient absorption. Despite efforts to reduce the incidence of worm infections, the problem is still prevalent in Indonesian children. Research conducted in Jakarta showed that >10% of children were infected with worms (Surja et al., 2021). Worm infections cause nutrients from the food that should be absorbed and used by the body to be absorbed and used for the growth and reproduction of worms living in the intestines (Gabain et al., 2023). Furthermore, worm infections are usually linked to poor personal hygiene and environmental sanitation, especially in densely populated urban areas (Gizaw et al., 2018).

Bandar Lampung is attractive as a research location. In addition to its high stunting incidence, several sub-districts are densely populated with poor sanitation. The author suspects that helminthic infections in Bandar Lampung are still high because there is still open defecation among the residents of Bandar Lampung (Yulyani et al., 2021). But, there is limited information on the adequacy of variation in animal protein intake and children's compliance with deworming consumption regarding stunting in Indonesia. Therefore, this research aims to analyze the relationship between the adequacy of variation in animal protein intake and consumption of dewormings with stunting in Bandar Lampung.

## 2 Method

### 2.1 Ethical statement

This research obtained ethical clearance from the Health Research Ethics Committee of Malahayati University at Lampung, Indonesia, and has been issued an ethical clearance letter with the number: 2921/EC/KEP-UNMAL/XI/2022.

### 2.2 Design and participants

This quantitative observational analytic research used a cross-sectional approach to analyze the relationship between adequate animal protein intake, deworming consumption, and stunting in under-fives in Bandar Lampung in 2022.

The sample included under-fives aged 12–59 months from 10 priority stunting handling neighbourhoods in Bandar Lampung, with two representations from neighbourhoods, namely Way Gubak Subdistrict, Sukabumi Regency, and Karang Maritim Subdistrict, Pajang Regency. The sample in this study was 262 under-fives. This is the total sample of under-fives who came to integrated service posts in selected neighbourhoods between November and December 2022.

The sampling technique used was purposive sampling, with inclusion criteria: under-fives living in Way Gubak and Karang Maritime neighbourhoods, under-fives living with their parents, under-fives and their mothers willing to be the sample in this study and good health. In comparison, the sample exclusion criteria in this study were under-fives with congenital disabilities, incomplete health records for under-fives at the integrated service post and under-fives who came to the integrated service post not with their mothers, for example, with caregivers or aunts.

### 2.3 Variable and data collection

The research used the occurrence of stunting as the dependent variable, which was measured directly by height and compared with z-scores. The cut off point for determining to stunt was a z-score of <−2 SD, with under-fives below this value categorized as stunting and those above as not stunting. The independent variables were completeness of basic immunization status, vitamin A consumption, deworming consumption, history of exclusive breastfeeding, and adequacy of animal protein intake (consumption habits of eggs, fish/meat, and milk).

The completeness of the basic immunization status variable was assessed from the documentation of the immunization history of the under-five and interviews with the mother of the under-five. If an under-five gets all the basic immunizations: BCG, Polio 1–4, DPT 1–3, HB 1–4 and MR immunization, then the immunization status is categorized as complete, but if there were immunizations that the under-five did not get, then the immunization status was classified as incomplete (Ministry of Health of the Republic of Indonesia, 2017a).

The variable consumption of vitamin A tablets is categorized as consuming if every six months consumes vitamin A following the recommendations from the government (Firmansyah, 2020). Conversely, if an under-five does not consume vitamin A every six months, he is categorized as not consuming vitamin A regularly.

The deworming consumption variable was measured by asking the mothers of under-fives. Has the mother given her child dewormed medicine every six months according to government recommendations? If an under five consumes worm medicine every six months, it is categorized as taking it regularly. Conversely, if an under-five does not periodically take deworming medication every six months, then he is classified as not taking deworming medication regularly (Ministry of Health of the Republic of Indonesia, 2017b).

When the baby only consumes breast milk for the first six months of life, it is categorized as getting exclusive breastfeeding. Conversely, if it is not only breast milk but other food/drinks that are also given, it is classified as not receiving exclusive breastfeeding (The Republic of Indonesia Government Regulation No 33, 2012).

The variable adequacy of variations in animal protein intake in under-fives is measured directly by asking the under-fives mother. In the past week, has your under-five consumed: eggs, fish/meat, and drank milk? If an under five consumes all three, then the under five is categorized as consuming quite a variety of animal protein. Meanwhile, if the last week, an under five did not consume everything (eggs, fish/meat, & milk), then his animal protein intake is categorized as less in variety.

The characteristics of the mothers were also observed educational level, employment status, parity, age during pregnancy, history of consuming iron tablets during pregnancy and adolescence, history of Antenatal Care (ANC) during pregnancy, pregnancy status, birth attendant, and duration of maternal smartphone use, which were obtained through questionnaires.

### 2.4 Statistical analysis

The data collected were tabulated and analyzed to determine the relationship between the independent and dependent variables using chi-square and Fisher's exact tests and logistic regression analysis aided with SPSS version 25.

## 3 Results

Table 1 showed that 16% (42 under-fives) were stunted, 20.6% of under-fives were not given deworming, and 19.8% did not consume enough food containing animal protein (eggs, fish/meat, milk). There were still 15.6% of under-fives who were not given exclusive breastfeeding as babies, and 42 (16%) did not get additional vitamin A. Furthermore, 19.1% of under-fives did not receive complete basic immunization.

**Table 1:** Results of bivariate analysis.

Variable	Stunting		Total	%	OR	pValue
	Stunted	Normal			CI	
**Consumption of Deworming:**						
No	31	23	54	20.6	24.1	0.001⁎
Yes	11	197	208	79.4	10.7–54.3	
**Animal Protein Intake (eggs, fish/meat, milk)**						
Less Vary						
Quite Varied	25	27	52	19.8	10.5	0.001⁎
	17	193	210	80.2	5.03–21.9	
**Exclusive Breastfeeding:**						
No	14	27	41	15.6	3.57	0.002⁎
Yes	28	193	221	84.4	1.67–7.63	
**Consumption of Vitamin A:**						
No	25	17	42	16	17.5	0.001⁎
Yes	17	203	220	84	7.96–38.7	
**Basic Immunization:**						
Incomplete	20	30	50	19.1	5.75	0.001⁎
Complete	22	190	212	80.9	2.80–11.8	

⁎ *p* < 0.05, the difference was statistically significant.

Table 1 shows that 16% (42 under-fives) experienced stunting, and 20.6% of under-fives were not given deworming drugs. Almost all mothers reasoned that they did not give deworming drugs because they did not come to the integrated service post when deworming was given. 19.8% of children under five need to consume more variety of foods containing animal protein (eggs, fish/meat, milk). Most children under five no longer drink milk (67%) and rarely eat meat because the meat is relatively expensive.

There are still 15.6% of children under five who were not exclusively breastfed when they were babies, with the most reason being that breast milk was not produced in sufficient quantities. Only 42 under-fives (16%) did not get additional vitamin A. The most common reason was mothers and under-fives who did not come to the integrated service post when vitamin A was given.

Furthermore, 19.1% of children under five still need complete basic immunization. Most under-fives do not get DPT immunization (88%) because there is an assumption that DPT immunization causes children to have a fever. This was why the mother of the under-five did not allow DPT immunization to be given to her child. In contrast, the most complete immunization given to under-fives is polio immunization.

Table 1 also showed that there was a relationship between stunting incidence in under-fives and consumption of deworming (*p* = 0.001), adequacy of animal protein intake (p = 0.001), history of exclusive breastfeeding (*p* = 0.002), consumption of additional vitamin A (0.001), and completeness of immunization base (0.001).

To fully understand the stunting incidence of under-fives in Bandar Lampung, mothers' characteristics of these children must be described as presented in Table 2.

**Table 2:** Characteristics of Mothers.

Variable	Stunting		Total	%	pValue	OR
	Normal	Stunted				CI 95%
**Mother's Education**						
Basic (Elementary School & Junior High School)	72	15	87	33.3	0.706	
Middle & High School (Minimum Senior High School)	148	27	175	66.7		
**Parity:**						
Primipara	65	16	81	30.9	0.272	
Multipara	155	26	181	69.1		
**Mother's Occupation:**						
Work outside the home	7	0	7	2.7	0.516	
Housewife	213	42	255	97.3		
**Age at Pregnancy:**						
Not at Risk (between 20 and 35 years old)	181	31	212	81	0.201	
At risk (<20 years or >35 years old)	39	11	50	19		
**Mother's Height:**						
Normal (150 cm and above)	196	24	220	83.1	0.001⁎	6.12
Short (under 150 cm)	24	18	42	17.9		2.91–12.8
**History of Consumption of Mother's Fe Tablets as Adolescents**						
Yes						
No	79	16	95	36.6	0.803	
	140	26	167	63.7		
**History of Iron Tablet Consumption during Pregnancy**						
Yes						3.487
No	187	26	213	81.3	0.001⁎	1.69–7.19
	33	16	49	18.7		
**Pregnancy Status**						
Planned	209	27	236	90	0.001⁎	10.55
Unplanned	11	15	26	10		4.39–25.3
**ANC History**						
Regular (min 4 times)	207	32	239	91.2	0.001⁎	4.97
Irregular (less than 4 times)	13	10	23	8.8		2.01–12.2
**Helper During Childbirth**						
Health workers	208	30	238	90.8	0.001⁎	6.93
Non-Health Workers	12	12	24	9.2		2.85–16.8
**Duration of Mother's Smartphone Use**						
Not Using a Smartphone	65	10	75	28.6	0.019⁎	
<2 h/day	118	20	138	52.7		
>2 h/day	25	24	49	18.7		

⁎ *p* < 0.05, the difference was statistically significant.

Maternal height, history of consumption of iron tablets during pregnancy, pregnancy status, history of ANC, birth attendant and duration of use of the mother's smartphone were significantly related to the incidence of stunting in under-fives. Meanwhile, the mother's education level, parity, employment status, age during pregnancy, and history of table iron consumption as a teenager were not significantly related to the incidence of stunting in under-fives (Table 2).

Table 3 showed that the most dominant variable with a significant relationship to stunting incidence was the history of deworming consumption (*p* = 0.001) and adequacy of animal protein intake (*p* = 0.002).

**Table 3:** Multivariate analysis results.

Variable	B	Sig	OR	CI (95%)
Consumption of deworming	2.11	0.001⁎	8.24	2.76–24.5
Consumption of animal protein intake	1.44	0.002⁎	4.24	1.66–10.8
Exclusive breastfeeding	0.84	0.108	2.33	0.83–6.57
Consumption of vitamin A	0.85 0.34	0.138 0.536	2.34 1.41	0.76–7.21 0.47–4.19

⁎ *p* < 0.05, the difference was statistically significant.

## 4 Discussion

Table 1 showed that 42 (16%) under-fives still experienced stunting in the research location. This result was similar to the stunting survey by SSGI in Lampung in 2022, which reported a prevalence of 15.8% (Khoiriyah, 2022). This value has yet to reach the target of below 14%, which will be achieved next year. Based on Table 2, administering deworming to under-fives is significantly associated with stunting in under-fives in Bandar Lampung (*p* = 0.001). Worm infections were shown to reduce the number of nutrients that can be absorbed by the body, as parasitic worms use the nutrients consumed by children for their growth and reproduction (Gabain et al., 2023). Children who suffer from worm infections often experience anemia, which can hinder their growth (Gujo and Kare, 2021). Worm infections also increased in densely populated areas and those with poor sanitation. Worm infections are often associated with an increased incidence of iron deficiency anemia(Sharan et al., 2018), as iron is the raw material for forming haemoglobin.

Adequacy of animal protein source intake was significantly associated with stunting in under-fives in Bandar Lampung (p = 0.001). Nutrient content in animal protein food was higher quality than in plant-based sources. The animal protein was more easily digested and contain more essential amino acids necessary for growth (Day et al., 2022). However, food sourced from animal protein is usually more expensive, making it less accessible to the broader community. Recent research also reported a linear relationship between the consumption of animal protein and increased height in children(Kaimila et al., 2019).

A history of exclusive breastfeeding was also significantly associated with stunting in under-fives in Bandar Lampung (*p* = 0.002). The complete nutritional content and presence of antibodies in breast milk were believed to maintain the growth and health of infants who received exclusive breastfeeding and can prevent stunting (Sari et al., 2021).

Consumption of vitamin A was significantly related to stunting incidence in under-fives at Bandar Lampung (*p* = 0.001). According to Wang et al. (2021), vitamin A is essential in improving children's immune systems, hence, less susceptible to recurring infectious diseases, thereby it can prevent stunting. Research in Uganda also showed a strong correlation between vitamin A deficiency and an increased incidence of stunting in children (Ssentongo et al., 2020).

The completeness of basic immunization in under-fives was significantly related to the stunting incidence in Bandar Lampung (p = 0.001). Basic vaccination is necessary to prevent children from contracting preventable infectious diseases, as healthy children are less likely to suffer from physical conditions associated with sickness. This is because children with infectious diseases are at a higher risk of experiencing stunting (Hendraswari et al., 2021).

The characteristics of mothers in this research, as presented in Table 2, showed that 66.7% had Elementary and Junior High School education, while only 33.3% or 87 mothers had at least Senior High School education. A previous report has established that education will significantly affect the mother's thinking maturity and parenting style toward the child (Margot Jackson et al., 2017). Research conducted in Ethiopia concluded that the level of mothers' education is an essential factor associated with stunting occurrence in children (Amaha and Woldeamanuel, 2021). This is because mothers with higher education will obtain better exposure to child nutrition information than those with low education. Educated mothers can also better absorb health information (Husnaniyah et al., 2020). They also have better employment opportunities and earn additional income, which can be used to improve the food budget for their families.

The results also revealed that 17.9% of mothers had short stature, <150 cm. The statistical test results show a significant relationship between maternal stature and the incidence of stunting in under-fives (*p* = 0.001) with an OR value of 6.11. These results indicate that mothers with short stature are 6.11 times more likely to have stunted under-fives than mothers with tall stature (>150 cm). Genetic and environmental factors also influence the child's height, but nutrition during pregnancy and growth also significantly impacts the child's size (Jelenkovic et al., 2016). Therefore, optimizing environmental factors such as nourishment is necessary and can affect the final height outcome. Child growth can be optimized by improving the quality of nutrition for the child or after the baby is born (Salleh et al., 2021).

Approximately 63.7% of respondents did not receive iron tablets during adolescence, while 81.3% received and consumed iron/Fe tablets during pregnancy. The statistical test results showed that giving iron tablets during pregnancy was significantly related to the incidence of stunting in under-fives (p = 0.001), with an OR of 3.48. These results indicate that pregnant women who do not take iron tablets during pregnancy will be 3.48 times at risk of having stunted under-fives compared to mothers who regularly consume iron tablets during their pregnancy. Providing iron tablets to pregnant women in Indonesia aims to reduce the incidence of anemia, which remains prevalent. Anemia in pregnant women can increase the risk of the disease in children and contributes to the increasing cases of stunting in children (Gaston et al., 2022). However, iron tablet provision is not recommended for pregnant women who do not have anemia, as it can lead to fetal growth cessation (Hwang et al., 2013).

The result indicated that 10% of children were born from unplanned pregnancies. Pregnancy planning is also crucial for a mother's physical and psychological preparation. However, when the pregnancy is unplanned, mothers often experience many health problems, including inadequate nutrition intake, lack of iron tablets, depression, and a desire for pregnancy termination (Ermiati et al., 2022), which can significantly affect fetal growth.

Table 2 revealed that 8.8% of mothers did not receive regular ANC during pregnancy. The frequency and regularity of ANC visits and the implementation of ANC service standards are significantly associated with stunting prevention (Wahyuni et al., 2021). The quality provision of ANC services by professional healthcare workers is one of the government's efforts to address many pregnancy-related issues. However, there still needs to be more variation in the education and competency of ANC service providers. The distance between the pregnant mother's residence and healthcare facilities also affected the regularity of ANC visits (Steinbrook et al., 2021).

Approximately 9.2% and 32.6% of mothers gave birth without the assistance of healthcare workers, as found in this research and the 2017 Indonesia Demographic and Health Survey, respectively (Tari and Andriani, 2022). Although this condition can occur due to several factors (Kumbani et al., 2013), the risk of health problems during and after childbirth is still very high.

Babies born to professional healthcare workers receive standardized services, immunization, and better health education. Based on the results of the Basic Health Survey conducted in 2018, it was reported that birth attendants were significantly associated with the occurrence of stunting in children in Indonesia (Simbolon et al., 2021). There are strong family and cultural factors that affect decision-making regarding birth attendants. The low bargaining position of mothers prevents them from making decisions, and tends to follow the recommendation of their family members (Aryastami and Mubasyiroh, 2021).

This research also revealed that 28.6% of mothers did not use smartphones, while 18.7% used the device for >2 h daily. This smartphone usage behaviour is suspected to affect the time used to care for their children, including preparing and providing nutrition. The more time spent on smartphone use by mothers, the less time there is to prepare food (Maqfiro et al., 2021). However, the positive effects of the information exposure gained by mothers who use smartphones should also be considered. This is because smartphone users have the opportunity to receive information from the internet about nutrition for their children and family, which will influence their decision-making (Moon et al., 2019).

Based on the multivariate test using logistic regression, it was found that the two most dominant variables related to stunting in under-fives at Bandar Lampung were consumption of deworming (*p* = 0.001) with an OR of 8.24 and adequacy of animal protein intake variation (*p* = 0.002) with an OR value of 4.24 (Table 3). Consuming dewormings can reduce the risk of children getting worm infections, allowing optimal nutrient absorption without parasite interference. This indicated that ensuring under-fives regularly take dewormings every six months is essential to prevent worm infections and stunting. Improving personal hygiene and environmental cleanliness is also necessary for avoiding worm infections. Meanwhile, various foods containing animal protein, such as eggs, fish/meat, and milk, are excellent for fulfilling the nutritional needs of children's growth. Animal proteins have been proven to contain more complete nutrients, which are easier to digest and absorb by the body compared to plant-based protein foods (Day et al., 2022).

Since 2017, the Government of Indonesia has had regulations in implementing worm infection eradication programs by administering deworming drugs to children over one year of age. This deworming program is carried out every six months. However, many children in Indonesia still do not participate in this program. Several reasons have been reported: the uneven distribution of worm medicine and the limited staff running this program (Febriyanti and Idris, 2020). Poor environmental sanitation and low socio-economic conditions in several regions make Indonesia vulnerable to worm infections. Even 195 million people risk being infected with soil-transmitted helminth (STH) throughout Indonesia (Tan et al., 2014). The behaviour of urban communities in Indonesia who defecate in the open is one of the influencing factors. The main reason is the lack of a toilet, especially for migrants who do not have permanent residence (Yulyani et al., 2021). Plus, most under-fives don't want to take deworming tablets because the size of deworming tablets is relatively large and has an unpleasant taste.

In early 2023, the Indonesian government is campaigning for the consumption of animal protein to reduce stunting (Directorate of Community Promotion and Empowerment, M. of H. of the R. of I, 2023). Previous studies reported a relationship between milk consumption and the incidence of stunting in under-fives (Sjarif et al., 2019). However, the consumption of animal protein in Indonesia is still lower than in Malaysian (Khusun et al., 2022). Residents in urban areas consume more chicken and beef, while residents in rural areas consume more fish because it is cheaper than beef. The amount of family income, educational level, and socio-cultural influence the amount of animal protein consumption in the family (Khusun et al., 2022).

An alternative solution to reduce stunting is to make sure children want to take the deworming drug regularly. It is necessary to try to replace the deworming tablet preparations with a liquid form with a sweet taste so that children are more comfortable and easy to consume. Communities also need to be encouraged to optimize the potential of local food sourced from fish. Indonesia is an archipelagic country rich in food potential from fish, but its potential has not been optimized. Intake of animal protein from fish in large quantities will be able to substitute for the lack of variation in animal protein intake from other food sources, although this still requires further research.

The characteristics of mothers under-five also need to be considered in preventing stunting because several maternal characteristics, such as maternal height, consumption of iron tablets during pregnancy, pregnancy status, history of ANC, helper during childbirth, and duration of mother's smartphone use, have been proven to be related to the incidence of stunting in under-fives in Bandar Lampung.

The limitation of this research is the relatively short interview time when meeting under-fives and their mothers who are only at the integrated service post. As a result, not all under-five data can be studied completely. Some mothers of under-fives also do not carry their under-fives development logbook, so the data only relies on information from mothers of under-fives.

## 5 Conclusion

The most dominant factor related to stunting in under-fives at Bandar Lampung in 2022 is the history of deworming consumption (*p* = 0.001) and sufficient variation in animal protein intake (*p* = 0.002). The results showed the need for regular dewormings of children and improved personal hygiene for both mothers and children to prevent worm infections. Efforts are also required to increase animal protein intake, especially from local food sources in the surrounding environment. Mothers of under-fives are needed to be educated about stunting prevention, especially in the regular administration of deworming and the importance of sufficient and varied animal protein intake.

## Declaration of Competing Interest

The authors declare that they have no known competing financial interests or personal relationships that could have appeared to influence the work reported in this paper.
